# Synaptic plasticity in infralimbic cortex mediates socially-acquired nicotine self-administration

**DOI:** 10.1186/1471-2105-16-S15-P1

**Published:** 2015-10-23

**Authors:** Hao Chen, Tengfei Wang, Wenyan Han

**Affiliations:** 1 Department of Pharmacology, University of Tennessee Health Science Center, Memphis, TN38163, USA

## Background

Social environment plays a critical role in the initiation of cigarette smoking among adolescents. It has been established that nicotine, the principal psychoactive ingredient of tobacco products, has both aversive and rewarding properties. Using olfactogustatory stimuli as the sensory cue for intravenous nicotine self-administration, we have shown that social learning of nicotine contingent odor cue prevented rats from developing conditioned taste aversion (CTA) and allowed them to instead establish stable nicotine self-administration. We also have shown that infralimbic cortex is a critical brain region for the effect of social learning in reversing nicotine conditioned aversion. We hypothesized that gene expression changes related to synaptic plasticity in infralimbic cortex underlies this effect of social learning.

## Materials and methods

We trained rats to self-administer intravenous nicotine in either an inducing or a neutral social environment, with contingent olfactogustatory cues for three days. Rats were then tested using a standard CTA protocol on day four. Rats were killed immediately after the CTA test and infralimbic cortex was dissected. RNA was then extracted for transcriptome sequencing using the Ion Proton instrument. Reads were mapped to the reference genome (rn6). Gene expression levels were estimated using HTSeq. The RefSeq database was used as the reference. DESeq2 was then used to normalize the expression levels and to identify statistically significant differences between the groups placed in the inducing and neutral social environments.

## Results

Gene set enrichment analysis (GSEA) supported our hypothesis that genes related to synaptic plasticity are preferentially regulated by the social environment.

